# Cultural attributes of suicidal ideation among older immigrants: a qualitative study

**DOI:** 10.1186/s12877-021-02628-6

**Published:** 2022-02-17

**Authors:** Mengting Li, Stephanie Bergren, Melissa Simon, XinQi Dong

**Affiliations:** 1 Institute for Health, Health Care Policy and Aging Research, Rutgers, The State University of New Jersey, 112 Paterson Street, 2nd Floor, New Brunswick, NJ 08901 USA; 2 School of Nursing, Rutgers, The State University of New Jersey, Newark, NJ 07102 USA; 3 Department of Obstetrics and Gynecology, Feinberg School of Medicine, Northwestern University, Chicago, IL 60611 USA

**Keywords:** Suicidal ideation, Immigrant, Minority

## Abstract

**Background:**

Suicide is a large and growing public health problem. Little is known about the attributes of suicidal ideation (SI) in the contexts of immigration. This qualitative study aims to explore immigration- and acculturation-related attributes of SI among older immigrants.

**Methods:**

A qualitative semi-structured interview design. Interview were conducted with 57 older Chinese immigrants in Chicago with self-reported SI in the past month.

**Results:**

In addition to attributes of SI which have been well documented in the literature, we identified immigration- and acculturation-related attributes, including linguistic and cultural barriers of being integrated to the receiving communities, acculturation gaps in intergenerational support, and uselessness.

**Conclusions:**

Findings of the study highlight the intersectionality of race, culture, and aging regarding SI, which are essential to improve recognition and understanding of SI among immigrant populations.

**Supplementary Information:**

The online version contains supplementary material available at 10.1186/s12877-021-02628-6.

## Introduction

Suicide was the 10th leading cause of death for all ages in the United States and approximately one person died by suicide every 11 min in 2019 [1]. Suicidal behaviors (SBs) are classified into three categories: suicidal ideation (SI), suicide plan, and suicide attempt (SA) [2]. SI refers to thoughts of engaging in behavior intended to end one’s life [2]. Evidence has shown that 90% of unplanned and 60% of planned first suicide attempts occurred within 1 year of the onset of SI [3]. Continued efforts are needed to increase outreach to untreated individuals with SI before the occurrence of attempts and to improve treatment effectiveness for such cases [4]. Globally, 9.2% of adults have lifetime SI, and women have a higher risk of SI than men [5]. Prevention of suicide may be improved by enhancing the understanding of SI. A study showed that 77% of those who committed suicide visited at least one health professional 3 months prior to death [6]. Research on the etiology of SB may help health professionals identify individuals at a high risk of suicide and connect them with relevant prevention and intervention programs.

Different age groups are associated with different risks for suicide. In the United States, suicide is the second leading cause of death for people aged 10–34, the fourth leading cause among people aged 35–44, the fifth leading cause among people aged 45–54, and the eighth leading cause among people aged 55–64 in 2019 [1]. Suicide rates among older adults declined substantially over the twentieth century, which may be owed to the improved social welfare system, better access to healthcare services, and effective treatments for depression [7]. Existing research on SI focus on adolescents and young adults, while older adults have received little attention [8, 9]. The risk factors for suicide vary across age groups. The younger the age at suicide, the higher the likelihood of increased comorbidity, particularly with personality and substance disorders [10]. Potential attributes of SI and behaviors among older adults include social isolation, family problems, loneliness, stressful life events, and physical illness [11, 12]. Existing research on SI of older adults focused on the group with life-limiting conditions, such as terminal cancer [13, 14]. The extant literature suffers from a lack of research examining SI among an immigrant population, older immigrants in particular. Older immigrants from ethnic minority populations (e.g., U.S. Chinese older adults) have linguistic and cultural barriers in the receiving country and heavily rely on their adult children to have access to healthcare and social services. Their social networks predominantly consist of family members, and they are isolated from the community [15]. Their perceived burdensomeness to children and their social isolation may lead to SI. In addition, despite significant progress made through epidemiological research on SI, the majority of the prior research is quantitative, and there is a dearth in the literature understanding the subjective experiences of older adults with SI in sociocultural contexts [16].

Suicide rates also vary by race/ethnicity, with the highest rates among non-Hispanic American Indian/Alaska Native, followed by non-Hispanic White populations [1]. However, SI has been understudied in racial and ethnic minority populations. Among these populations, existing mental health disparities might be exacerbated by institutional barriers faced by them. Asian Americans have lower rates of using mental health services than other populations, which might be due to stigma associated with mental illness and linguistic and cultural barriers in using healthcare services [17]. According to the estimated number of Asian Americans in 2017, the Chinese population (5.2 million) constitute the largest segment of the Asian American population [18]. Depression is the principal risk factor for SI in late life [19, 20]. The prevalence rates of depressive symptoms in community-dwelling older Chinese Americans range from 17.1 to 28% [21, 22], which is substantially higher than the prevalence rates reported in older Americans (4.9 to 16%) [23, 24]. Given the relatively high prevalence rates of depressive symptoms in older Chinese Americans, their risks of SI may also be high. Meanwhile, little is known about the attributes of SI among older Chinese immigrants. Research on attributes of SI could inform interventions to reduce SI in this high suicide risk population. Guided by the interpersonal theory of suicide, this study will examine the attributes of SI among U.S. Chinese older adults.

### Theoretical framework

The interpersonal theory of suicide proposes two important psychological states as attributes of SI: 1) thwarted belongingness (unmet needs for social connectedness and belongingness) and 2) perceived burdensomeness (the perceptions of burdensomeness to others) [25–27]. The theory asserts that when people hold the two psychological states in their minds simultaneously, and when they do so for long enough, they develop the desire for death [28].

Thwarted belongingness is what people feel they are alienated from others, not an integral part of family and friend networks, or other valued groups. Thwarted belongingness is a multidimensional construct, comprised of loneliness and absence of reciprocal care (ones in which individuals both feel cared about and demonstrate care of another). Thwarted belongingness has been highlighted in multiple suicide theories and plays a central role in the etiology of SB. According to Durkheim (1987), dysregulation of social forces, degrees of social integration in particular, results in suicide [29]. Perceived burdensomeness is the view that “my death will be worth more than my life to family, friends, and society” [28]. Perceived burdensomeness is comprised of liability and self-hate. The relationships between thwarted belongingness, perceived burdensomeness, and SI have been tested in diverse populations, including young adults [28], adults [30], psychiatric patients [31], older adults [32, 33], and immigrants (see a review [34]).

With emerging theoretical frameworks on the etiology of SI, more empirical studies among minority immigrant populations are needed to reflect the attributes of SI in an immigration context for better prevention and intervention strategies. This study aims to use a qualitative approach to understand the attributes of SI among U.S. Chinese older immigrants with recent SI.

## Method

### Sample

The participants of the seed program were recruited in conjunction with community partner organizations and outreach through the Population Study of Chinese Elderly (PINE). The PINE Study is a representative study of Chinese older adults in Chicago, and it screens for recent SI. One goal of ​the seed program is to understand suicidal ideation and behaviors in U.S. Chinese older adults. The recruitment criteria of the seed program include: 1) self-identified as Chinese, 2) age 50 and above, 3) SI was evaluated by one item “thoughts that you would be better off dead, or of hurting yourself in some way in the past month”), and 4) no significant cognitive impairment with Mini-Mental State Examination (MMSE) score > 20. The eligibility for the seed program was screened between 2014 and 2018 among PINE participants. Eighty one older adults were eligible for the seed program and fifty seven of them consented to participate in this program. The institutional review board at Rush University approved this study.

### Data collection

In order to maximize cultural and linguistic sensitivity of this investigation, a semi-structured interview guide was developed by the investigative team. The review of the suicide literature, evidence-based practice, and the interdisciplinary team (e.g., psychologists, sociologists, clinicians, and community partners) provided the justification and rationale to develop the interview guide of merit and worth with implications for improving practice regarding U.S. Chinese older adults with SI. Core research questions of the interview guide included attributes of SI, maladaptive and adaptive coping with SI, and barriers to help-seeking behaviors (Additional file 1: Appendix).

As SI is a sensitive topic in Chinese communities and stigma is associated with SI among this population, we took a culturally relevant approach to interview participants with SI. In the interview guide, we de-emphasized the participant’s own SI to prevent the participant from feeling uncomfortable or blamed. Instead, we started with a broad conversation on SI in Chinese communities, and the participants were willing to share with their own life stories and SI. All intervention sessions and evaluations were conducted in private settings by the preferred language and dialect of participants (e.g., Mandarin, Cantonese).

Interviews were conducted by research assistants (RAs). RAs were jointly trained by the investigative team through an in-depth orientation and by our academic institution and community partners through training sessions. RAs training focused on the research protocol, approaching sensitive questions, framing culturally relevant questions, etc. The investigative team further conducted mock interviews and in-depth interviews for RAs prior to field interviews, ensuring standardization of the protocol and training in relevant cultural issues embedded in the research.

To monitor quality of our RAs, our research team conducted random visits to observe RAs on 1) ability to communicate; 2) knowledge and understanding of the interview questions; 3) understanding of the protocol; and 4) understanding of the community and cultural settings. In addition, audio of the interviews was reviewed for quality and RAs were given feedback on further probing. RAs who had areas for improvement received additional training by the interdisciplinary team.

### Data analysis

Descriptive analyses (proportions) characterized study participants. Audio recordings were transcribed for analysis. Two bilingual coders used an integrated approach of grounded theory (deductive) [35] and thematic (inductive) techniques [36–39] to analyze the transcripts. In initial coding, one coder identified observable themes guided by theoretical constructs pertaining to our key research question.

Another coder contrasted and revised the initial codes against the theoretical constructs and original data to improve comprehensiveness. The coders also used thematical analysis techniques to identify themes emerging from the transcripts that are beyond existing constructs. Disagreements in coding were discussed and resolved among all investigators and to reach consensus on a final codebook. Last, the two coders selected representative quotes from the transcripts and translated independently. The coders reached consensus on a final version of translation, which was verified by another bilingual research assistant. All transcripts were analyzed using Nvivo 12.

## Results

Table 1 presents sociodemographic characteristics of the 57 U.S. Chinese older adults with past-month SI. Among participants, 37 (64.9%) are women, 42 (73.7%) were over 70 years old, and 41 (73.2%) had less than 13 years of education. Many (59.6%) were married and most (71.9%) were living with at least one person. Most (83.9%) had been to the United States for more than 10 years. Tables 2 and 3 summarize key qualitative findings and representative quotations.

**Table 1:** Sociodemographic Characteristics of US Chinese Older Adults Participants with Past-Month Suicidal Ideation

Participant Characteristics		
Age, N (%)	50–69	17 (29.8%)
	70–79	25 (43.9%)
	80+	15 (26.3%)
Sex, N (%)	Men	20 (35.1%)
	Women	37 (64.9%)
Education, years N (%)	0–6	13 (23.2%)
	7–12	28 (50.0%)
	13+	15 (26.8%)
Marital status, N (%)	Married	34 (59.6%)
	Single/Divorced/Widowed/Never married	23 (40.4%)
Number of people in the household, N (%)	0	2 (3.5%)
	1	11 (19.3%)
	2	22 (38.6%)
	3+	22 (38.6%)
Country origin, N (%)	Mainland China	49 (87.5%)
	Others	7 (12.5%)
Years in the US, N (%)	1–10	9 (16.1%)
	11–20	21 (37.5%)
	21–30	16 (28.6%)
	31+	10 (17.9%)

**Table 2:** Thwarted Belongingness from Qualitative Interviews with US Chinese Older Adults with Past-month Suicidal Ideation

Organizing Themes	Basic Themes	Conceptual meanings	Translation
Social isolation		Lack of social connections and feel loneliness	
	Linguistic and cultural barriers of being integrated to the receiving communities^a^	Linguistic barriers and cultural gaps	I’m lonely so that it’s easy for me to have this thought (suicidal). Because I have nothing, I’m lonely here, especially at night. I don’t speak the language here.
	Low social support^b^	Perceived low social support from family and friends	Because people who are closest to me have to work, they don’t come often. I can’t cook and can’t walk. My daughter is busy. She has her own family. Nobody can help me. All of my friends are old. How can they help?
	Self-reported loneliness^b^	Feeling lonely and disconnected to others	I feel very lonely...My son and daughter live far from me. When they didn’t come to see me, I feel lonely.
	Restricted social networks^b^	The absence of marriage or small numbers of children and friends.	I don’t like Chicago since I moved from New York one or two years ago. In New York, I had many friends who get along well and all speak Cantonese. I have to start over and adapt to the new environment. I don’t want to make friends here, because I only understand 2–3 out of 10 sentences they talk.
	Seasonal reductions in social interactions^b^	Season-caused reduction in social interactions	I can’t go outside for activities when it’s cold. I’m bored staying home.
Absence of reciprocal care		Not a supporter of others and have few to no support	
	Conflict in family norms^b^		You can hardly expect someone filial enough to persist care of long-ill parent (“*jiu bing chuang qian wu xiao zi*”). My children expect me to die soon; then they can get the money. I lost the hope to rely on the children. I often think why not to die soon. I’m in poor health. I cause trouble for children.
	Acculturation gaps in intergenerational support^a^	A sense of imbalance in the relationship and the violation of social expectations due to acculturation gaps	My daughter-in-law looks down upon me. What is your (my) worth? You (I) can only clean and take care of the grandchildren. Initially, they ask us to go there, and we take care of grandchildren. They seem to take it for granted. In the US, American grandparents do not have to take care of their grandchildren. If you want them to, you must pay them money.
	Loss through death^b^		I have been suffering since my wife was gone. My daughter has two kids and her husband. It doesn’t matter. But for me, I don’t have anyone. When I feel painful and bored, I leave the senior apartment and I took the train, from the beginning to the very end, and from the end back to the beginning.

^a^ Immigration- and acculturation-related novel attributes experienced by immigrants; ^b^ Attributes experienced by both immigrants and non-immigrants and reshaped by immigration

**Table 3:** Perceived Burdensomeness from Qualitative Interviews with US Chinese Older Adults with Past-month Suicidal Ideation

Organizing Themes	Basic Themes	Conceptual meanings	Translation
Liability		Feel their death is worth more than their life to others	
	Illness and Functional Impairment^b^	Distress from serious physical illness; such as medical comorbidity, self-reported health	Because I get ill often, (I often) think, would it be nice if people die without knowing it? This thought contradicts to suicide. I hope my eyes don’t open by the time I wake up tomorrow. My life would pass, and (so do) the awful things.
	Family Burden^b^	Belief that one is a burden on family	They (Chinese older adults in the US) feel being alive is to bring troubles to children. I agree with it; (being alive) is to add burden to others. Older people have no (potentials for) development.
	Expendability and being unwanted by children^b^	Feel expendable, unwanted	My eldest son doesn’t help me. When I was young, he asked me to work for him. He was afraid that I would leave. Now that I am old and useless, he wants to kick me away.
Self-hate			
	Self-blame, shame^b^	Attributing adversities to self	I think my fate and personality hurt myself. So, I’m unhappy now, and I can’t tell anybody.
	Low self-esteem^b^	Low self-esteem	Being angry, having stomach problems, and my physical strength is not as good as before…I feel like ten years older. I used to feel strong, and I can do anything. Now, I have little interest in doing things. I realize that I am old. This has diminished my confidence greatly, and all my hopes are shattered.
	Uselessness^a^	Feel useless and inability regarding social participation	Being old is useless, and I want to die if there is nothing I can do. It is meaningless to be alive.

^a^ Immigration- and acculturation-related novel attributes experienced by immigrants; ^b^ Attributes experienced by both immigrants and non-immigrants

### Thwarted belongingness

Based on the narratives, we regard thwarted belongingness as the unmet need to belong. Thwarted belongingness includes “social isolation” and “an absence of reciprocal care” (Table 2). Social isolation is categorized by a lack of contact with others. Social isolation is multidimensional, manifesting by restricted social networks (structural), low social support (functional), seasonal reductions in social interactions (behavioral), low integration to the receiving communities (cultural), and perceived loneliness (emotional). During interviews, having *low social support* was frequently mentioned in this population. As described by one participant, “My daughter is busy. She has her own family. Nobody can help me. All my friends are old. How can they help?” *Loneliness* was commonly reported. One participant felt “very lonely”, and “My son and daughter live far from me. When they didn’t come to see me, I feel lonely”. *Linguistic and cultural barriers of being integrated to the receiving communities* was mentioned as potential attributes of psychological distress and SI among this immigrant population. One participant reported “I’m lonely so that it’s easy for me to have this thought (suicidal). Because I have nothing, I’m lonely here, especially at night. I don’t speak the language here.” Other participants reported “I don’t speak English. I will get lost if I go outside by myself. I have limited social engagement”; “I can’t adapt to the life here. I don’t speak English. I have no friend”; and “The life in the US is like a birdcage. The door is closed. The window is closed. I watch TV at home. I don’t see anybody”. Other themes aligning with the theoretical constructs of thwarted belongingness are *restricted social networks*, manifesting by small numbers of children and friends, and *seasonal variation*.

An absence of reciprocal care describes the absence of reciprocal relationships in which individuals both feel cared about and provide care to others. *Conflicts in family norms* appear as one manifestation of the absence of reciprocal care. An older participant referred to a Chinese proverb meaning “one can hardly expect someone filial enough to persist care of his long-ill parent” to describe strained relationships with the children that might have been exacerbated by the older participant’s declining health conditions. *Acculturation gaps in intergenerational support* was identified in this population. Chinese culture emphasizes interdependence between generations, while American culture values independence. Immigrants are affected by both traditional culture and receiving culture. Different levels of acculturation in aging parents and adult children could lead to gaps in intergenerational support. Some participants described the feeling of not being connected or understood with children and grandchildren because of different life experiences and acculturation levels among generations of immigrants: “They (son and daughter-in-law) ask us to go there, and we take care of grandchildren. They seem to take it for granted. In the US, American grandparents do not have to take care of their grandchildren. If you want them to, you must pay them money.” A few participants revealed *loss through death*.

### Perceived burdensomeness

Perceived burdensomeness describes the beliefs that one is so flawed that one’s existence is a burden on family, friends, and society (“liability”) and affectively laden cognitions of self-hatred (“self-hate”; Table 3). With respect to liability, psychological distress causes by *illness and functional impairment* and perceiving themselves as *family burden* were frequently mentioned. One older adult described a passive suicidal thought because of chronic physical illness; “Because I get ill often, (I often) think, would it be nice if people die without knowing it? This thought contradicts to suicide. I hope my eyes don’t open by the time I wake up tomorrow. My life would pass, and so do the awful things.” Some Chinese older adults also think of themselves as burden to others: “Older people have no (potentials for) development.” Some participants expressed *expendability and being unwanted by children*.

Regarding self-hate, older adults *blamed themselves*; one said, “I think my fate and personality hurt myself”. Some participants appeared *low self-esteem*. Additionally, expression of feeling *useless* emerged among Chinese older adults. Immigrants feel useless in the host country. One thinks that “Being old is useless” and “It is meaningless to be alive”.

## Discussion

From the perspectives of U.S. Chinese older adults with past-month SI, this exploratory qualitative study identified potential attributes of SI among this population. Findings of the study highlight the intersectionality of race, culture, and aging regarding SI, which are essential to improve recognition and understanding of SI among this population.

Our findings provide empirical evidence for the interpersonal theory of suicide hypothesizing that experiencing facets of thwarted belongingness and perceived burdensomeness are observable among older adults with SI. The findings also expanded the theory by discovering novel potential attributes of SI specific to the aging minority population, including linguistic and cultural barriers of being integrated to the receiving communities, acculturation gaps in intergenerational support, and perceived uselessness.

One prior qualitative study found that family support and interpersonal connectedness are strong factors increasing meaning in life in suicidal patients, and particular emphasis was placed on the protective role of intergenerational relationships [40]. Building upon existing evidence of the close relationship between family support and SI, our findings suggested that acculturation gaps in intergenerational support in the context of immigration might be an underlying cause of SI among older Chinese immigrants. Younger immigrants generally are more acculturated to the mainstream culture in the receiving communities compared to their parents [41]. In Chinese immigrant families, adult children are more prone to the individualistic values of the host culture, whereas their parents are more likely to retain traditional collectivist values and endorse high filial expectation [42, 43]. If adult children are unable to meet the filial expectation of aging parents, older adults are likely to have depressive symptoms, which may result in SI [42, 44].

Consistent with prior studies [28, 34], we found that restricted social networks are a potential attribute of SI. A population-based large-scale study has shown that older Chinese immigrants experience linguistic barriers and restricted social networks in the United States [15, 45]. The restricted social networks in the migration contexts may explain the high prevalence of depressive symptoms in this immigrant population [21, 22]. Additionally, our study provides evidence that restricted social networks may result in SI in this population. Interventions aimed at promoting mental health and reducing SI of older Chinese Americans could reduce their linguistic and cultural barriers and help them to be better integrated to the receiving communities.

Bronfenbrenner’s ecological theory highlights the micro-, meso-, exo-, and macrosystems in which an individual embedded [46]. Family systems and neighborhood contexts represent the microsystem that constitutes the immediate environment in which individuals actively engage in social relations [47]. Our findings support the developmental/system theories that attribute SB to disturbed family systems [48, 49] and social forces [29], the microsystem of an individual’s ecosystem. Immigration has profound effects on the microsystem of immigrants, reshaping their family and social relationships. The dysfunctional microsystem in receiving communities may trigger immigrants’ SB. The macrosystem is the broad ideological values and norms of a particular culture. Our study reveals that the host culture (macrosystem) may influence family and social relationships (microsystem), and in turn lead to SB.

In Berry’s acculturation model, immigrants are divided into four groups: separation (high attachment to culture of heritage and low attachment to the host culture), assimilation (low attachment to culture of heritage and high attachment to the host culture), integration (high attachment to culture of heritage and high attachment to the host culture), and marginalization (low attachment to culture of heritage and low attachment to the host culture) [50]. Our study shows that older adults with low attachment to the host culture (i.e., linguistic and cultural barriers of being integrated to the receiving communities) may have a high risk of SB. However, it remains unclear whether the attachment to the culture of heritage is a risk or protective factor for SB. According to Berry’s acculturation framework, some immigrants with low attachment to the host culture could have high attachment to culture of heritage (separation), while others have low attachment to both the host culture and culture of heritage (marginalization). Future research could explore the relationship between separation or marginalization and SB.

These results should be interpreted with caution. First, the findings may not be able to generalize to older Chinese immigrants in other geographical areas or other ethnic groups. Second, the themes identified in this qualitative study are potential attributes of SI. Future quantitative studies are needed to understand the underlying mechanism of SI. Third, the seasonal suicide is related to the geographical area of the study site. The winter is long in Chicago and restricts social engagement of older adults. Seasonal suicide may not be shown in the suicide research in other geographical areas. Fourth, risk factors may differ in studies adopting different definitions for SI. In our study, SI was evaluated by the question “In the past month, how often have you been bothered by thoughts that you would be better off dead, or of hurting yourself in some way”, which represents a range from nearly every day to only once in the past month. It is necessary to pay attention to the definitions of SI when comparing different suicide studies.

Despite these limitations, this study has important theoretical and practical implications. This study connects the interpersonal theory of suicide with an immigration context. Immigration- and acculturation-related attributes were added to the interpersonal theory of suicide. The interpersonal theory of suicide outlines how dysfunctional microsystem invokes SB. Our study advances the interpersonal theory of suicide by identifying that both the microsystem and macrosystem could exert an effect on SB.

This study might inform interventions by identifying immigration- and acculturation-related attributes of SI in immigrant populations. Linguistic and cultural barriers in the receiving community and different paces in acculturation across generations within the family may lead to restricted social networks, low social support, loneliness, uselessness, and family conflicts, which are the attributes of SI. Prevention and intervention programs could help older immigrants to be better integrated to the receiving community to reduce their SI. Social service could target immigrant families to reduce intergenerational conflict to prevent suicide and achieve healthy aging. This study presents a novel framework that outlines immigration- and acculturation-related attributes of SI for researchers and healthcare professionals interested in addressing the complicated but critical problem of SB in racial and ethnic minority aging populations.

## Supplementary Information

**Additional file 1: Appendix.** Interview Guide.

## Data Availability

The datasets used and/or analyzed during the current study are available from the corresponding author on reasonable request.
