# Supplementary material for: Cultural attributes of suicidal ideation among older immigrants: a qualitative study
Source: BMC Geriatr. 2022 Feb 17;22(Suppl 1):678. doi: 10.1186/s12877-021-02628-6 (PMC8851697; doi:10.1186/s12877-021-02628-6)
Supplement: Supplementary file 1 — Additional file 1: Appendix. Interview Guide. [file 12877_2021_2628_MOESM1_ESM.docx]

#### Appendix 1.

#### Interview Guide

**Perception on Mental Illness and Suicide**

1. What have you heard about suicide? How common is it in our community vs other racial/ethnic groups?
2. What do you think causes suicidal thought among Chinese older adults?

**Behavioral Changes**

1. What are the similarities between Western treatments and Chinese treatments for mental illness? Differences?
2. If you get angry/sad/frustrated/disappointed/etc, or overwhelmed with negative emotions, what do you do as a response?
   1. What are the consequences of that response?
   2. What type of help would see you seek?
   3. What activities do you do that impact your mood? How often do you do these activities?
   4. When you think about these activities that you have done, what about them makes you continue to do them?

**Facilitators and Barriers to Help**

1. Why would Chinese older adults suffering from mental illness not seek help?
2. What do you think are potential behavioral changes that can help ameliorate mental illness?
3. What are the expectations of family members to help care for someone suffering from a mental illness?
   1. How do/can Chinese families help care for someone suffering from a mental illness?
4. What are the consequences/burdens/challenges that family members face when they help someone with a mental illness?
   1. Probing
      1. Impact on family relationships
      2. Emotionally
